# Quiet Quitting in the Hospital Context: Investigating Conflicts, Organizational Support, and Professional Engagement in Greece

**DOI:** 10.3390/nursrep15020038

**Published:** 2025-01-24

**Authors:** Aikaterini Toska, Ioanna Dimitriadou, Constantinos Togas, Eleni Nikolopoulou, Evangelos C. Fradelos, Ioanna V. Papathanasiou, Pavlos Sarafis, Maria Malliarou, Maria Saridi

**Affiliations:** 1 Department of Nursing, University of Thessaly, 41500 Larissa, Greece; ktoska@uth.gr (A.T.); efradelos@uth.gr (E.C.F.); iopapathanasiou@uth.gr (I.V.P.); psarafis@uth.gr (P.S.); malliarou@uth.gr (M.M.); msaridi@uth.gr (M.S.); 2 Department of Psychology, Panteion University of Social and Political Sciences, 17671 Athens, Greece; togascostas@yahoo.gr; 3 General Hospital of Argolida, 21200 Argos, Greece; sep2237sp@go.uop.gr

**Keywords:** quiet quitting, workplace conflicts, hospital management, organizational support, organizational justice

## Abstract

**Background/Objectives:** Quiet quitting, defined as employees fulfilling only the minimal requirements of their roles without extra effort or engagement, poses unique challenges in high-stress environments like hospitals where commitment directly impacts patient care. This study investigates the phenomenon of “quiet quitting” within the healthcare sector, with a specific focus on hospital staff in Greece. **Methods:** A cross-sectional design was employed, surveying 186 healthcare professionals from the General Hospital of Argos using the Questionnaire for Conflicts in Healthcare Organizations and the Quiet Quitting Scale (QQS). **Results:** Descriptive and inferential statistical analyses revealed that 62% of participants exhibited characteristics of quiet quitting, with “lack of motivation” scoring highest (M = 2.80, SD = 0.987) among QQS subscales. Significant correlations were observed between perceived reward fairness and motivation levels (r = −0.194, *p* < 0.01) and between management awareness of contributions and both motivation (r = −0.313, *p* < 0.01) and initiative (r = −0.192, *p* < 0.01). Logistic regression identified perceptions of management awareness as a key predictor of quiet quitting (*p* < 0.05). **Conclusions:** The findings emphasize the critical role of equitable reward systems and managerial recognition in reducing disengagement. Strategies to enhance employee engagement and resolve workplace conflicts are essential for fostering a resilient healthcare workforce.

## 1. Introduction

In recent years, the concept of “quiet quitting” has emerged as a notable workplace phenomenon, especially in settings with high demands and intense workloads. Quiet quitting refers to a situation where employees perform only the minimum requirements of their job description, refraining from any extra effort, innovation, or contribution beyond the bare minimum [1,2]. While this behavior is minor, it can have significant effects, particularly in industries like healthcare where employee commitment is crucial. In hospital settings, where patient care and safety are paramount, the implications of quiet quitting are far-reaching, potentially affecting the quality of care, staff morale, and organizational performance [3,4].

The healthcare sector, and hospitals in particular, presents a unique work environment characterized by high levels of stress, long working hours, grueling shifts, emotional stress and limited resources. Due to these conditions, there has been a trend of disengagement of staff and the emergence of quiet quitting. Quiet quitting can have a negative impact on key areas of the hospital environment, including the quality of patient care, treatment efficiency and overall healthcare delivery, in addition to employee happiness [5,6,7]. To successfully address and reduce the impact of quiet quitting cessation, hospital management must be able to understand the underlying causes and prevalence of this phenomenon in their organizations [8].

Recent studies have specifically examined the effects of varying shift lengths on nurse-reported outcomes, revealing that extended working hours and overtime can negatively impact patient care continuity by reducing handover times and diminishing necessary shift overlap [3,9,10]. A cross-sectional survey in the United States measuring nurse burnout rates and examining factors associated with leaving or considering leaving employment due to burnout found that among nurses who reported leaving their job, 31.5% cited burnout as a reason and reported a stressful work environment and inadequate staffing [11]. In Greece, a study by Galanis et al. investigated the phenomenon of “quiet quitting” among nurses and its influence on turnover intentions. The findings were significant, with 60.9% of nurses identified as quiet quitters and 40.9% reporting high levels of turnover intention [12]. Importantly, the study demonstrated a positive correlation between higher levels of quiet quitting and increased turnover intention, highlighting the potential consequences of this emerging workplace trend in the nursing profession.

While quiet quitting itself is an emerging concept, its roots are intertwined with well-established factors such as workplace conflict, organizational support, and reward imbalance. For instance, workplace conflicts—often stemming from communication breakdowns, role ambiguity, or inequitable authority structures—have been shown to increase stress and disengagement among healthcare professionals [13,14]. Similarly, perceptions of inadequate organizational support play a critical role in shaping employee motivation and initiative. Employees who feel undervalued or unsupported by management are more likely to exhibit disengagement behaviors [15]. Reward imbalance further compounds these issues, with studies highlighting that perceived unfairness in promotions, financial rewards, or recognition significantly undermines employee morale and commitment [16,17].

Although substantial research has been conducted on general employee disengagement and burnout within the healthcare sector, the specific phenomenon of quiet quitting remains under-explored in the context of hospital staff. Previous studies have tended to focus on overt forms of employee dissatisfaction, such as turnover and absenteeism, without fully addressing the more covert, but equally detrimental, behavior of quiet quitting. The aim of this study is to investigate how workplace conflicts and organizational support influence quiet quitting among healthcare professionals in Greek hospitals. It examines the relationship between workplace conflicts, organizational support, and employee disengagement, while also considering the influence of demographic variables such as age, gender, and years of service. Our hypotheses were the following:

**H1.**  *Higher levels of perceived workplace conflict will be positively associated with quiet quitting behaviors*.

**H2.**  *Lower levels of perceived organizational support will be positively associated with quiet quitting behaviors*.

**H3**  *Employees who perceive rewards as unfairly distributed will exhibit higher levels of quiet quitting behaviors*.

## 2. Methods

### 2.1. Study Design

This study employed a cross-sectional design to investigate the attitudes of staff at a regional hospital in Greece regarding workplace conflicts and quiet quitting. It was consistent with the guidelines for reporting observational studies, the Strengthening the Reporting of Observational Studies in Epidemiology (STROBE) Statement.

### 2.2. Participants

From December 2023 to January 2024, we recruited 184 participants using convenient sampling. The study population comprised health professionals aged 18 to 67 years who were employed at the General Hospital of Argos in Greece. Participants were eligible for inclusion in this study if they: (1) were currently employed at the Nursing Unit of the General Hospital of Argos; (2) were over 18 years old; and (3) were willing to complete the questionnaire. Exclusion criteria included (1) inability to comprehend the questionnaire; and (2) history of mental or physical illness that could potentially influence responses. Demographic characteristics collected included age, gender, marital status, educational level, occupation, employment status, and managerial position. The sample size of participants was determined to ensure robust statistical power for detecting relationships between variables of interest in this cross-sectional study. Aiming for a minimum power of 0.80 with an alpha level of 0.05, the sample size was based on anticipated effect sizes derived from prior studies examining similar constructs in healthcare contexts.

### 2.3. Insrtuments

#### 2.3.1. Questionnaire for Conflicts in Healthcare Organizations

This instrument was developed specifically to measure sources of conflict in the workplace. Originally designed by Tengilimoglu and Kisa (2005) [18], the questionnaire has been adapted for use in various healthcare contexts, including Greece. It comprises five sections and includes a total of 25 items. These sections investigate the causes of workplace conflicts, organizational factors that may precipitate conflicts, group behavior during conflicts, and potential strategies for conflict resolution. Each item is rated on a Likert scale, allowing for both detailed and aggregate analyses of conflict-related factors.

#### 2.3.2. The Quiet Quitting Scale (QQS)

This is a recently developed instrument for evaluating quiet quitting among employees. It consists of nine items divided into three subscales:

- Detachment (items 1, 2, 3, 4), e.g., “I take as many breaks as I can”;
- Lack of initiative (items 5, 6, 7), e.g., “I don′t express opinions and ideas about my work because I am afraid that the manager assigns me more tasks”;
- Lack of motivation (items 8, 9), e.g., “I find motives in my job”.

The scale utilizes a 5-point Likert scale (1 = totally disagree−5 = totally agree or 1 = never−5 = always). Items 7, 8, 9 are reverse scored, and there is a total score as well as a score for each subscale. Employees with a QQS score ≥ 2.06 are classified as quiet quitters, while those with a QQS score <2.06 are classified as non-quiet quitters (Galanis et al., 2024) [19]. The scale has demonstrated robust psychometric properties (reliability and validity) in its original validation (Galanis et al., 2023). In the present study, Cronbach′s Alpha coefficient values were 0.669 for Detachment, 0.628 for Lack of initiative, 0.798 for Lack of motivation, and 0.760 for the total scale.

### 2.4. Procedures

This study employed structured paper-and-pencil questionnaires, administered during participants′ work shifts. Researchers distributed the questionnaires in person, providing participants with a brief explanation of the study′s purpose and instructions for completion. Completed questionnaires were collected directly to ensure data integrity. Participants were informed about the confidentiality of their responses and provided written consent before participation.

### 2.5. Data Analysis

Descriptive statistics were calculated to describe demographic characteristics and summarize response to QQS and the Questionnaire for Conflicts in Healthcare Organizations. Categorical variables were presented with frequencies and percentages, whilst quantitative variables were presented with mean, standard deviation, minimum, maximum, and range. Reliability analysis was performed using the Cronbach′s Alpha coefficient of each subscale of QQS. Differences in the Quiet Quitting scale and its subscales were examined with the *t*-test for independent samples or with One-Way ANOVA (Analysis of Variance). Possible differences between categorical variables with two levels were examined by the χ^2^ statistical criterion. Pearson′s and Spearman′s correlation coefficients were employed to examine correlations between workplace, conflict scores, organizational support perceptions and the subscales of the QQS. Additionally, a binomial logistic regression analysis with the backward elimination model was performed to further investigate the variables that distinguish those who had quietly quit work from those who had not. In this instance, the classification of the participants (YES, have quit/NO, have not quit) was utilized as the dependent variable. Statistical significance was set at 5% (*p* = 0.05). Finally, three multiple regression analyses were performed to examine how demographic and work-related characteristics predicted Detachment, Lack of initiative and Lack of motivation. To maintain data integrity and reduce bias, missing data were addressed using multiple imputation techniques, where feasible, to estimate missing values based on observed patterns in the dataset. Cases with significant missing responses that compromised their validity (e.g., more than 20% missing across key variables) were excluded from the analysis. Data analysis was conducted utilizing SPSS, version 29.

### 2.6. Approval Statement/Ethics Statement

This study was approved by the Scientific Council of the General Hospital of Argolida-Hospital Unit of Argos (*n*: 2325/23.11.2023) and by the 6th Regional Health Authority of Peloponnese, Ionian Islands, Epirus and Western Greece (*n*: 74429/06.12.2023). Participants were informed about the purpose of this study, their rights as participants, and the confidentiality of their responses prior to providing informed consent to participate.

## 3. Results

This study comprised a total of 186 participants, with a response rate of 54%. A total of 350 questionnaires were distributed, of which 186 were returned. Most participants were female (66.7%, *n* = 124), while males constituted 33.3% (*n* = 62). Regarding marital status, most participants were married with children (62.4%, *n* = 116), followed by single without children (22.0%, *n* = 41). The largest occupational group in the sample was nurses/midwives (24.2%, *n* = 45), followed by administrative staff (22.0%, *n* = 41), and physicians (12.9%, *n* = 24). Most participants held permanent positions (60.2%, *n* = 112), while 32.8% (*n* = 61) were employed on a temporary or fixed-term basis. The demographic and professional characteristics of participants are presented in Table 1.

Table 2 summarizes the participants′ concerns regarding workplace conflicts. A significant portion of participants (36.8%) believed that educational differences “much” contribute to communication problems between professional groups, while 33.5% felt that this factor moderately contributed. Furthermore, only 13% of participants reported receiving the rewards they felt their performance largely deserved, with 26.6% stating that they did not receive any rewards at all. When asked about the fairness of reward distribution, 30.8% of respondents believed there was no fairness at all, and only 1.1% felt there was very much fairness.

Participants provided various suggestions for resolving conflicts within the hospital (Table 3). The most frequently suggested solution was the fair distribution of authority (22.2%, *n* = 41). Other suggestions included reducing workplace politics (13.5%, *n* = 25) and ensuring a fair approach to reward and punishment (9.7%, *n* = 18).

QQS and its subscales are presented in Table 4. The mean score for “Detachment” was 2.18 (SD = 0.691), while the mean score for “Lack of initiative” was slightly higher at 2.40 (SD = 0.756). The highest mean score was for “Lack of motivation” at 2.80 (SD = 0.987), suggesting that this aspect of quiet quitting was more prevalent among the participants. Notably, 62% of the participants exceeded the threshold of 2.06, classifying them as quiet quitters.

Pearson and Spearman correlation coefficients were calculated to assess the relationships between the subscales of the QQS and participants′ perceptions of workplace factors (Table 5). A moderate positive correlation was observed between “Lack of initiative” and “Detachment” (r = 0.334, *p* < 0.01), while “Lack of motivation” demonstrated a strong correlation with “Lack of initiative” (r = 0.506, *p* < 0.01). Notably, perceptions of fair reward distribution were negatively correlated with “Lack of motivation” (r = −0.194, *p* < 0.01), suggesting that participants who perceived rewards as being distributed fairly were less likely to report a lack of motivation. Furthermore, hospital management′s awareness of participants′ contributions was negatively correlated with both “Lack of initiative” (r = −0.192, *p* < 0.01) and “Lack of motivation” (r = −0.313, *p* < 0.01), indicating that participants who perceived management as being aware of their contributions exhibited higher levels of motivation and initiative. Quiet quitting behaviors were observed across demographic groups. Female participants, who comprised 66.7% of the sample, reported higher tendencies toward disengagement behaviors. Similarly, permanent employees (60.2%) demonstrated notable quiet quitting tendencies, indicating that tenure security alone does not prevent disengagement.

A binomial logistic regression analysis was conducted to identify predictors of quiet quitting. The model incorporated variables such as gender, marital status, occupation, employment status, and participants′ perceptions of reward distribution, promotion opportunities, and management awareness. The results revealed that perceptions of management awareness significantly predicted quiet quitting (*p* < 0.05). Specifically, participants who perceived management as being unaware of their contributions were more likely to exhibit quiet quitting behaviors. The Nagelkerke R^2^ for the model was 0.24, indicating that approximately 24% of the variance in quiet quitting behaviors was explained by the predictor variables.

Moreover, three multiple linear regression analyses were conducted using the scores for Detachment, Lack of initiative and Lack of motivation as the dependent variable. These analyses revealed no indication of multicollinearity among the variables, as tolerance levels exceeded 0.1 and Variance Inflation Factor (VIF) values were below ten. The examination of Cook and Mahalanobis distance, Centered Leverage Value, and DfBetas and Dffits showed no evidence of outliers or influential points. The results of the multiple linear regression are given in Table 6. The results presented above indicate a statistically significant negative association of the conflicts with an administration employee and the conflicts with others with the score on Lack of motivation. This model was statistically significant (F = 2.651, *p* = 0.014), with the best predictor variable being the conflicts with others. The proportion of variance in the Lack of motivation score accounted for 15.5% (R = 0.499; R-squared = 0.249; Adjusted R-square = 0.155). No other variable predicted significantly the score on this dimension of quiet quitting, as well as on detachment and lack of initiative.

## 4. Discussion

Conflict is an inherent aspect of social and professional relationships that commonly arises in cooperative dynamics among individuals and groups. In the healthcare sector, this inevitability is exacerbated by the complex and high-stress environments in which healthcare workers operate. From front-line personnel, such as physicians and nurses, to supporting staff in administrative and technical roles, the potential for conflict is ever present [13,20]. The concept of “quiet quitting” in healthcare, though emerging as a critical topic, has been insufficiently explored in existing literature. While substantial research has focused on overt forms of workplace disengagement, such as burnout and turnover, covert behaviors like quiet quitting remain understudied, especially in hospital environments. This study addresses this gap by examining the organizational and interpersonal factors contributing to quiet quitting among hospital staff, with a particular focus on workplace conflicts and perceived organizational support.

The findings of this study present a significant prevalence of quiet quitting among hospital staff, particularly evident in the ’Lack of Motivation’ subscale, which recorded the highest mean score. Meanwhile, “Detachment” and “Lack of Initiative” also appeared in relation to levels but were comparatively less pronounced. These behaviors seem to be directly affected by organizational factors. More specifically, perceptions of unfair reward distribution were negatively correlated with “Lack of Motivation”. This means that employees who believed that rewards were distributed unfairly were less likely to find motivation in their work. Similarly, perceptions of insufficient management awareness of employee contributions were strongly associated with higher scores on ‘Lack of motivation’ and ‘Lack of initiative’.

Demographic data also provided information on the profile of workers most affected by quiet quitting. Female participants, comprising 66.7% of the sample, had higher disengagement tendencies, replicating findings in other studies where caring roles, typically dominated by women, face higher levels of stress in healthcare settings [21,22]. It is noteworthy that employees in permanent roles (60.2%) also reported significant quitting behaviors, possibly suggesting that tenure security does not necessarily moderate disengagement. Logistic regression analysis demonstrated that perceived management awareness significantly predicted silent cessation (*p* < 0.05). Employees who felt unrecognized by management were more likely to exhibit calm quitting behaviors, highlighting the importance of recognition and communication in promoting employee engagement.

Although there is limited research in the literature regarding this phenomenon, especially in health care, the findings of this study are closely aligned with the existing literature, especially Galanis et al., who similarly recorded high levels of quiet quitting among Greek nurses (60.9%) [12]. Recent research by Karadas and Çevik (2024) explored the psychometric properties of quiet quitting behaviors among Turkish healthcare professionals, highlighting that disengagement often correlates with perceived workplace injustice and communication barriers [6]. Both studies highlight lack of motivation as the most important dimension of disengagement, suggesting a systemic issue in the health sector. The strong associations found in this study between quitting behaviors and perceived unfairness in rewards echo broader organizational research. For example, studies of organizational justice have consistently shown that perceived inequities in reward systems reduce employee morale and increase disengagement [17,23]. The findings of this study, therefore, confirm the idea that fairness in tangible (financial) and intangible rewards (recognition) is a key point in maintaining employee engagement.

The higher scores for ’Lack of motivation’ and ’Lack of initiative’ suggest that quiet quitting in health care settings may manifest differently than other domains where disengagement is typically more evident, highlighting the role of intrinsic motivation in caring professions, where the work is deeply personal and emotionally demanding [15,24]. A recent observational study by Moisoglou et al. examined the impact of innovation support on quiet quitting, innovation behavior and innovation outcomes in 328 nurses [25]. Findings revealed that while most participants displayed characteristics of “quiet quitters”, including higher levels of disengagement, lack of initiative, and lack of motivation, they also displayed innovative behaviors. Furthermore, the study highlighted that providing strong support for innovation not only reduces the likelihood of quiet quitting but also encourages innovative behavior and enhances innovation outcomes.

The findings also revealed that ineffective conflict resolution strategies within the hospital significantly contribute to employee disengagement. Participants highlighted key solutions, such as implementing fair distribution of authority, minimizing workplace politics, and fostering professional management practices, all of which underscore a pressing need for systemic reform to address the underlying sources of discord. This aligns with Johansen et al. [14], who emphasize that unresolved conflicts, if not appropriately addressed, can exacerbate work-related stress, particularly among nurses. Without targeted interventions to address these issues, conflicts are likely to intensify, fostering an organizational culture marked by strained relationships, diminished morale, and disengagement [26].

However, this study also adds to the debate by emphasizing the role of conflict-related factors, such as poor communication and training disparities among staff, in exacerbating disengagement. About 36.8% of participants attributed significant communication barriers to educational differences, a new concept not extensively covered in previous research. Systemic challenges within the healthcare industry may also play a pivotal role, alongside organizational-level factors. Disparities in healthcare financing mechanisms, remuneration structures, workforce shortages, and divergent regulatory landscapes across countries can further amplify communication and training obstacles. For instance, healthcare systems with limited resources may experience exacerbated educational and training disparities due to inadequate nurse-to-patient ratios or insufficient funding for professional development, potentially leading to heightened employee disengagement [5].

Montgomery and Patrician (2022) [4] highlight the role of resilience-building programs and leadership training in reducing quiet quitting behaviors, particularly among nurse leaders navigating high-pressure environments. These findings align with the current study′s emphasis on managerial awareness and reward fairness, while also broadening the discussion to include strategies such as resilience training and enhanced leadership accountability. Incorporating these approaches into healthcare organizations could not only address quiet quitting but also foster a culture of engagement and innovation. By situating our findings within the broader context of these emerging studies, this research contributes to an evolving dialogue on effective interventions for mitigating quiet quitting in high-stress environments.

The limitations of this study primarily focus on its cross-sectional design, which restricts our ability to draw causal relationships. Although correlations between organizational factors were identified, longitudinal studies are necessary to establish causality and track changes in employee engagement over time. Additionally, this study′s focus on a single regional hospital in Greece limits the generalizability of its findings. While the results align with other Greek studies, the specific organizational culture and resource constraints of the hospital may not fully represent the diversity of healthcare settings, such as urban hospitals or private healthcare institutions. Also, the reliance on self-reported data introduces the potential for biases, including social desirability bias, where participants might under-report negative behaviors or over-report positive ones. Finally, the psychometric properties of the QQS, though validated in previous studies, exhibited moderate reliability for certain subscales within this sample (e.g., Cronbach′s alpha for Detachment = 0.669 and Lack of Initiative = 0.628). However, these measures were deemed appropriate and validated for use in this context.

Future research should focus on cohort studies to better understand the dynamics of quiet interruption behaviors and their underlying causes. Investigating additional factors such as leadership styles, emotional intelligence, and job satisfaction could provide a more comprehensive understanding of employee engagement. In addition, intervention studies, such as conflict resolution programs, enhanced reward systems, or management awareness initiatives, will provide useful insights into reducing silent quitting behaviors. Finally, examining the immediate impact of silent disruption on patient care outcomes will highlight the broader implications of this phenomenon, linking employee engagement to institutional performance and safety.

## 5. Conclusions

This study highlights the significant prevalence of quiet quitting among Greek hospital staff, emphasizing the role of organizational factors such as reward justice, conflict resolution, and management awareness in shaping employee engagement. Key limitations include the cross-sectional design and focus on a single site, limiting generalizability. Future research should explore causal relationships through longitudinal studies and examine interventions like resilience training and reward enhancements. Investigating the direct impact of quiet quitting on patient care outcomes would also be valuable. Addressing systemic drivers of disengagement, such as inequitable rewards and insufficient recognition, is critical for fostering a motivated and resilient healthcare workforce, ultimately improving both employee well-being and patient care outcomes.

## Figures and Tables

**Table 1:** Demographic and professional characteristics of participants (N = 186).

Characteristic	Frequency	Percentage (%)
Gender		
Male	62	33.3
Female	124	66.7
Marital/Relationship Status		
Single without children	41	22.0
Single with children	17	9.1
Married without children	10	5.4
Married with children	116	62.4
Occupation		
Physician	24	12.9
Nurse/Midwife	45	24.2
Nursing Assistant	24	12.9
Scientific Personnel	11	5.9
Paramedical Personnel	19	10.2
Other Personnel	14	7.5
Administrative Staff	41	22.0
Technical Staff	8	4.3
Employment Status		
Permanent	112	60.2
Specialty Training	7	3.8
Temporary/Fixed term	61	32.8
Internship	6	3.2
Managerial Position		
Yes	42	22.6
No	144	77.4

**Table 2:** Participants’ concerns about the factors causing conflict.

	Not at All (%)	Little (%)	Moderately (%)	Much (%)	Very Much (%)
How much do you think educational differences lead to communication problems between professional groups?	6.5%	14.1%	33.5%	36.8%	9.2%
Are your messages clearly understood and your job expectations shared by other professional groups?	5.4%	22.2%	41.6%	25.9%	4.9%
Do you receive the rewards you think your performance deserves (early promotion, financial gain, vacation, appreciation, etc.)?	26.6%	25.5%	31.5%	13%	3.3%
Do you think there is fair distribution of rewards across different professional groups?	30.8%	29.2%	29.2%	9.7%	1.1%
How much do you think hospital management is aware of your contribution to health service production?	20%	28.6%	33%	13.5%	4.9%
How much do your promotions and career advancements match your expectations?	10.3%	22.2%	32.4%	27%	8.1%

**Table 3:** Suggestions considered most important by participants for resolving conflicts in their hospital.

	N	%
Fair distribution of resources	2	1.1
Communication and coordination should be established in the organization	17	9.2
Causes of conflicts should be detected and both sides should be listened to	17	9.2
Fair approach to reward and punishment	7	3.8
Meetings should be held	8	4.3
No discrimination, management should be neutral	18	9.7
Distribution of authority should be made	41	22.2
Less workplace politics	25	13.5
Professional management should take control, departments should be autonomous	13	7
Respect to personal rights, occupational career	11	5.9
Fair wages	24	13
Other	4	2.2

**Table 4:** Descriptive statistics for the QQS and its subscales.

	N	Mean	Std. Deviation	Minimum	Maximum	Range
Detachment	185	2.18	0.691	1	5	4
Lack of initiative	185	2.40	0.756	1	5	4
Lack of motivation	185	2.80	0.987	1	5	4
Total score	184	2.39	0.588	1	4	3

**Table 5:** Correlations between QQS subscales and workplace factors.

	Detachment	Lack of Initiative	Lack of Motivation	QQS- Total Score
Lack of initiative	0.334 **	-	0.506 **	0.789 **
Lack of motivation	0.238 **	0.506 **	-	0.723 **
QQS-Total score	0.760 **	0.789 **	0.723 **	*-*
How much do you think educational differences lead to communication problems between professional groups?	−0.003	−0.082	−0.009	−0.042
Are your messages clearly understood and your job expectations shared by other professional groups?	−0.180 *	−0.091	−0.261 **	−0.232 **
Do you receive the rewards you think your performance deserves (early promotion, financial gain, vacation, appreciation, etc.)?	−0.010	−0.080	−0.264 **	−0.137
Do you think there is fair distribution of rewards across different professional groups?	0.030	0.003	−0.194 **	−0.061
How much do you think hospital management is aware of your contribution to health service production?	−0.108	−0.192 **	−0.313 **	−0.260 **
How much do your promotions and career advancements match your expectations?	−0.061	−0.117	−0.281 **	−0.192 **

Note: **. Correlation is significant at the 0.01 level (2-tailed); *. Correlation is significant at the 0.05 level (2-tailed).

**Table 6:** Multiple linear regression with the score in Detachment, Lack of initiative and Lack of motivation as the dependent variable.

*Dependent Variable = Detachment*								
							*95% CI*	
		Unstandardized Coefficients	Standard Error	Standardized Coefficients	t	*p*	Lower	Upper
M₀	(Intercept)	2.154	0.085		25.349	<0.001	1.985	2.324
M₁	(Intercept)	2.682	0.249		10.786	<0.001	2.186	3.179
	Gender (male vs. female)	−0.236	0.184		−1.280	0.205	−0.604	0.132
	Having received information about the conflicts (YES vs. NO)	−0.176	0.198		−0.888	0.378	−0.571	0.219
	Managerial Position (YES vs. NO)	−0.074	0.230		−0.323	0.748	−0.534	0.385
	Conflicts with a colleague (YES vs. NO)	0.207	0.298		0.694	0.490	−0.389	0.803
	Conflicts with Supervisors (YES vs. NO)	0.071	0.266		0.268	0.790	−0.459	0.602
	Conflicts with subsistent (YES vs. NO)	−0.092	0.260		−0.355	0.723	−0.611	0.427
	Conflicts with an administration employee (YES vs. NO)	−0.263	0.259		−1.016	0.313	−0.780	0.254
	Conflicts with others (YES vs. NO)	−0.278	0.269		−1.033	0.305	−0.816	0.260
***Dependent variable = Lack of initiative***								
M₀	(Intercept)	2.324	0.083		27.957	<0.001	2.158	2.490
M₁	(Intercept)	2.554	0.243		10.503	<0.001	2.068	3.040
	Gender (male vs. female)	−0.127	0.182		−0.700	0.486	−0.491	0.236
	Having received information about the conflicts (YES vs. NO)	−0.144	0.194		−0.743	0.460	−0.533	0.244
	Managerial Position (YES vs. NO)	0.181	0.225		0.802	0.425	−0.269	0.630
	Conflicts with a colleague (YES vs. NO)	0.161	0.298		0.542	0.590	−0.434	0.756
	Conflicts with Supervisors (YES vs. NO)	0.081	0.261		0.311	0.757	−0.440	0.602
	Conflicts with subsistent (YES vs. NO)	0.277	0.254		1.090	0.280	−0.231	0.786
	Conflicts with an administration employee (YES vs. NO)	−0.459	0.253		−1.813	0.075	−0.964	0.047
	Conflicts with others (YES vs. NO)	−0.352	0.277		−1.268	0.210	−0.906	0.203
***Dependent variable = Lack of motivation***								
M₀	(Intercept)	2.918	0.115		25.282	<0.001	2.688	3.148
M₁	(Intercept)	3.406	0.309		11.024	<0.001	2.789	4.023
	Gender (male vs. female)	−0.134	0.229		−0.586	0.560	−0.592	0.324
	Having received information about the conflicts (YES vs. NO)	0.254	0.246		1.034	0.305	−0.237	0.745
	Managerial Position (YES vs. NO)	0.011	0.286		0.037	0.971	−0.561	0.582
	Conflicts with a colleague (YES vs. NO)	0.570	0.371		1.538	0.129	−0.170	1.310
	Conflicts with supervisor (YES vs. NO)	−0.141	0.330		−0.426	0.671	−0.800	0.518
	Conflicts with subsistent (YES vs. NO)	0.401	0.323		1.243	0.218	−0.244	1.046
	Conflicts with an administration employee (YES vs. NO)	−0.846	0.322		−2.631	0.011	−1.488	−0.204
	Conflicts with others (YES vs. NO)	−0.882	0.335		−2.636	0.011	−1.550	−0.214

Note: Standardized coefficients can only be computed for continuous predictors.

## Data Availability

The data that support the findings of this study are available on request from the corresponding author.

## Public Involvement Statement

There was no public involvement in any aspect of this research.

## Guidelines and Standards Statement

This manuscript adheres to STROBE guidelines for observational studies.
